# Supplementary material for: The type VI secretion system governs strain maintenance in a wild mammalian gut microbiome
Source: bioRxiv. 2025 Dec 8:2025.11.29.690828. Preprint. [Version 3] doi: 10.1101/2025.11.29.690828 (PMC12694586; doi:10.1101/2025.11.29.690828)

bioRxiv preprint doi: https://doi.org/10.1101/2025.11.29.690828; this version posted December 8, 2025. The copyright holder for this preprint (which was not certified by peer review) is the author/funder, who has granted bioRxiv a license to display the preprint in perpetuity. It is made available under aCC-BY 4.0 International license.

Supplemental Figure 1. Evidence supporting stability of the WildR microbiome over multiple generations and identification of an ICE-encoded T6SS

(A) Schematic depicting the generation of the WildR F7 cryoprserved stocks from the combined cecal contents of six mice used to propagate the community, and subsequent community characterization steps. (B-C) Comparison of the abundance of the 100 genera most prevalent in wild donor mice between the indicated communities. Points are shaded to show overlapping datapoints (darker shades), and Spearman's correlation for each comparison is indicated (ρ). ND, not detected. (D) Mapping efficiency of metagenomic reads from different WildR community generations to selected murine-derived genome databases: the WildR catalog (86 MAGs and genomes from the WildR, generated in this study) or the comprehensive mouse microbiota genome catalog (CMMG; 1573 species across mouse microbiomes (Kieser et al. 2022)). (E) To-scale schematic of the ICE containing the GA1 T6SS encoded in *B. acidifaciens* and *B. caecimuris* F12. Location of single base deletion in *B. caecimuris* F12 highlighted in the orange box.

Supplemental Figure 2. Activity of the *B. acidifaciens* T6SS against species co-resident in the WildR community.

(A) *In vitro* mating efficiency of the integrative plasmid pNBU2-*ermG:: tssC* into *B. acidifaciens*. "RM silent" indicates plasmid was mutated to remove a *B. acidifaciens* methylated motif. Data shown are mean ± SD from 3 independent matings. N.D., not detected; D.L.,

bioRxiv preprint doi: https://doi.org/10.1101/2025.11.29.690828; this version posted December 8, 2025. The copyright holder for this preprint (which was not certified by peer review) is the author/funder, who has granted bioRxiv a license to display the preprint in perpetuity. It is made available under aCC-BY 4.0 International license.

detection limit. (B) Recipient abundance after *in vitro* growth competitions between *B. acidifaciens* donors lacking various structural components of the T6SS and the indicated recipient species. (C) Competitive index from *in vitro* growth competition between indicated WildR isolates (recipient) and *B. acidifaciens* donors. For B & C, data show the mean ± SD of technical replicates from one biological replicate and represent results from at least three biological replicates. *$p \leq 0.05$ by two-tailed t-test; all other comparisons were not significant. (D) *B. caecimuris* F5 abundance in cecal contents from germ-free mice co-colonized with *B. acidifaciens* wild-type or Δ*tssC*. Data show the mean ± SD and points indicate values from individual mice (n=6) across two biological replicates. *$p \leq 0.05$ (two-tailed t-test). (E) *P. vulguatus* abundance in feces from germ-free mice (n=6, two biological replicates) co-colonized with *B. acidifaciens* wild-type or Δ*tssC* and *P. vulguatus*. Boxplots represent the interquartile range with indicated mean for each condition, whiskers represent minimum and maximum values, points represent individual values. *$p \leq 0.05$ (two-way ANOVA with repeated measures test and Šidák's multiple comparisons test). (F) *P. vulguatus* abundance in cecal contents from mice described in panel E. Data show the mean ± SD and points show values from individual mice (n=6). No statistical difference was found based on *B. acidifaciens* genotype by two-tailed t-test. (G) Relative abundance (% reads per kb per million) of selected WildR Bacteroidales species in the WildR F7 generation. Data are mean + SD from cryopreserved WildR stocks and 2 fecal samples from mice used to propagate the WildR F7 community.

Supplemental Figure 3. Addition of *B. acid* ^{exo} to the WildR does not alter community composition and enables *in situ* measurement of T6SS-mediated fitness.

bioRxiv preprint doi: https://doi.org/10.1101/2025.11.29.690828; this version posted December 8, 2025. The copyright holder for this preprint (which was not certified by peer review) is the author/funder, who has granted bioRxiv a license to display the preprint in perpetuity. It is made available under aCC-BY 4.0 International license.

(A,B) Recovery of *B. acid* ^{exo} from feces (A) and cecal contents (B) following gavage of germ-free mice with the WildR community and the indicated amount of *B. acid* ^{exo} relative to the endogenous population. (C,D) Recovery of *B. acid* ^{exo} or *B. acid* ^{exo} Δ*tssC* from feces (D) or cecal contents (E) from mice colonized with the WildR and *B. acid* ^{exo} strains. *$p \leq 0.05$ (two-way ANOVA with repeated measures test and Šidák's multiple comparisons test in C, unpaired t-test in D). For data in panels A-D, boxplots represent the interquartile range with indicated mean for each condition, whiskers represent minimum and maximum values, and points show values from individual mice. (E) Principal coordinate analysis of weighted Unifrac diversity metrics calculated from 16S rRNA amplicon sequencing data from feces collected from mice colonized with the WildR alone or in combination with the indicated strain of *B. acid* ^{exo}. Gavage samples highlighted in pink and remaining timed fecal and cecal (collected at 56 days post gavage) samples are colored as indicated. Data shown are from one biological replicate, representative two experiments conducted.

Supplemental Figure 4. Distribution of the T6SS-ICE in the WildR suggests limited fitness benefit to some *Bacteroides* sp.

(A) Schematic of ICE-seq approach to identify WildR species encoding the ICE. The junction amplification and sequencing strategy applied to both ends of the ICE is depicted only for the 3′ end for simplicity. (B) Schematic depicting ICE transfer from *B. acidifaciens* (marked with Cm^R) to *P. vulgatus* (marked with Erm^R) via *in vitro* conjugation and selective plating. (C) To-scale schematic of ICE insertion sites in *P. vulgatus* ^{exo} + ICE transconjugants that acquired the indicated versions of the ICE. (D) Abundance (relative to total *P. vulgatus*) of the indicated *P. vulgatus* ^{exo} in fecal samples from mice co-colonized with the WildR community, as determined

bioRxiv preprint doi: https://doi.org/10.1101/2025.11.29.690828; this version posted December 8, 2025. The copyright holder for this preprint (which was not certified by peer review) is the author/funder, who has granted bioRxiv a license to display the preprint in perpetuity. It is made available under aCC-BY 4.0 International license.

by qPCR. Boxplots represent the interquartile range with indicated mean for each condition, whiskers represent minimum and maximum values, and points show values from individual mice (n=8) from two biological replicates. Asterisks indicate significant differences between *P. vul* ^{exo} + ICE and *P. vul* ^{exo} + ICE Δ*tssC* frequency at the indicated time points (p<0.05, Šídák's multiple comparisons test, mixed model ANOVA with multiple comparisons). N.D., not detected. (E) Principal coordinate analysis of weighted Unifrac diversity metrics calculated from 16S rRNA gene amplicon sequencing data from feces collected from mice (n=8/group across 2 biological replicates) colonized with the WildR and either *P. vul* ^{exo} + ICE (closed circles) or *P. vul* ^{exo} + ICE Δ*tssC* (open circles). The community composition varied between groups at early time points (purple; Rep 1, p=0.021, pseudo-F=2.7; Rep 2, p=0.011, pseudo-F=12, PERMANOVA test), but varied less or not signifcantly at late time points (orange; Rep 1, p=0.25, pseudo-F=1.3; Rep 2, p=0.003, pseudo-F=5).

bioRxiv preprint doi: https://doi.org/10.1101/2025.11.29.690828; this version posted December 8, 2025. The copyright holder for this preprint (which was not certified by peer review) is the author/funder, who has granted bioRxiv a license to display the preprint in perpetuity. It is made available under aCC-BY 4.0 International license.

Supplemental Figure 1

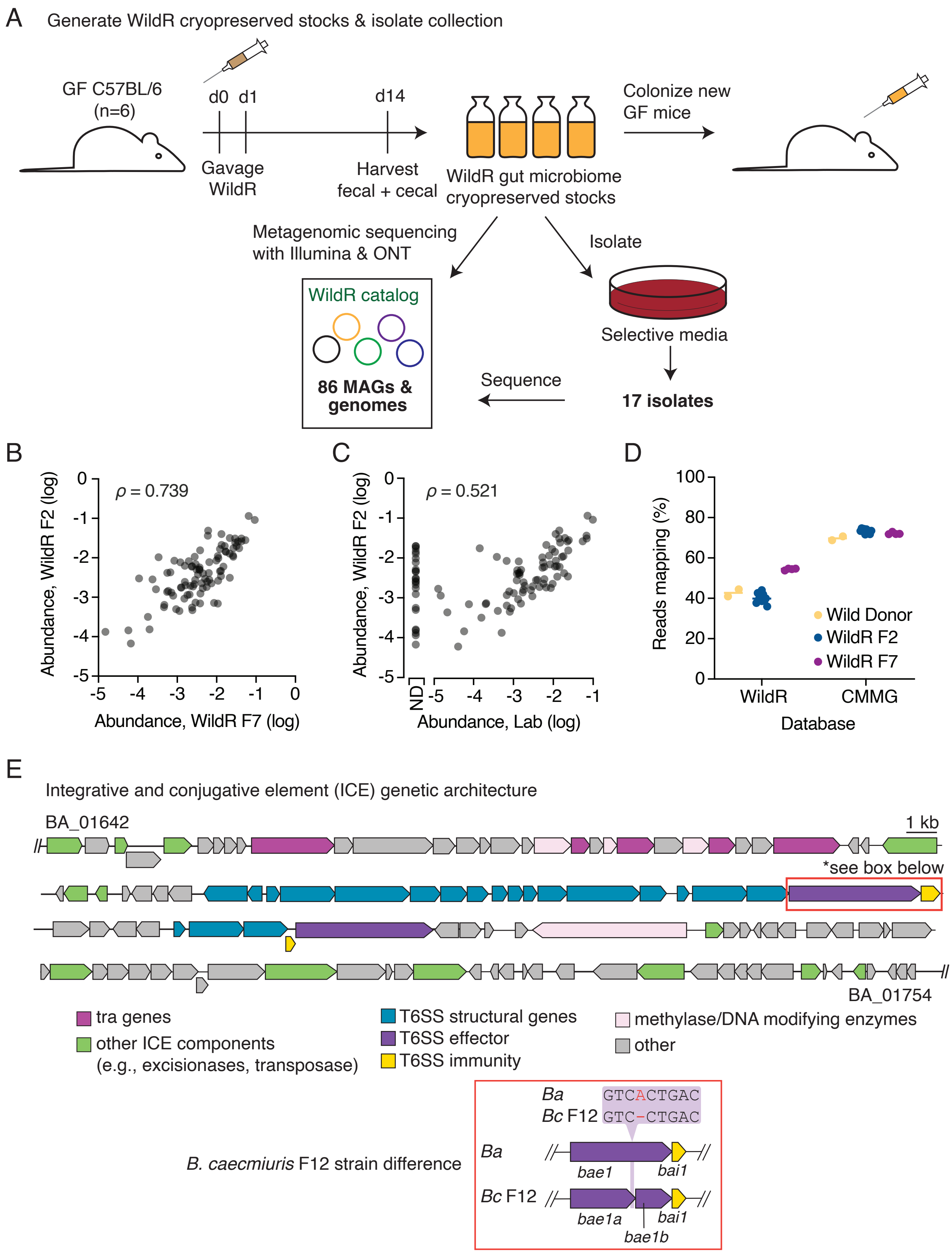

bioRxiv preprint doi: https://doi.org/10.1101/2025.11.29.690828; this version posted December 8, 2025. The copyright holder for this preprint (which was not certified by peer review) is the author/funder, who has granted bioRxiv a license to display the preprint in perpetuity. It is made available under aCC-BY 4.0 International license.

Supplemental Figure 2

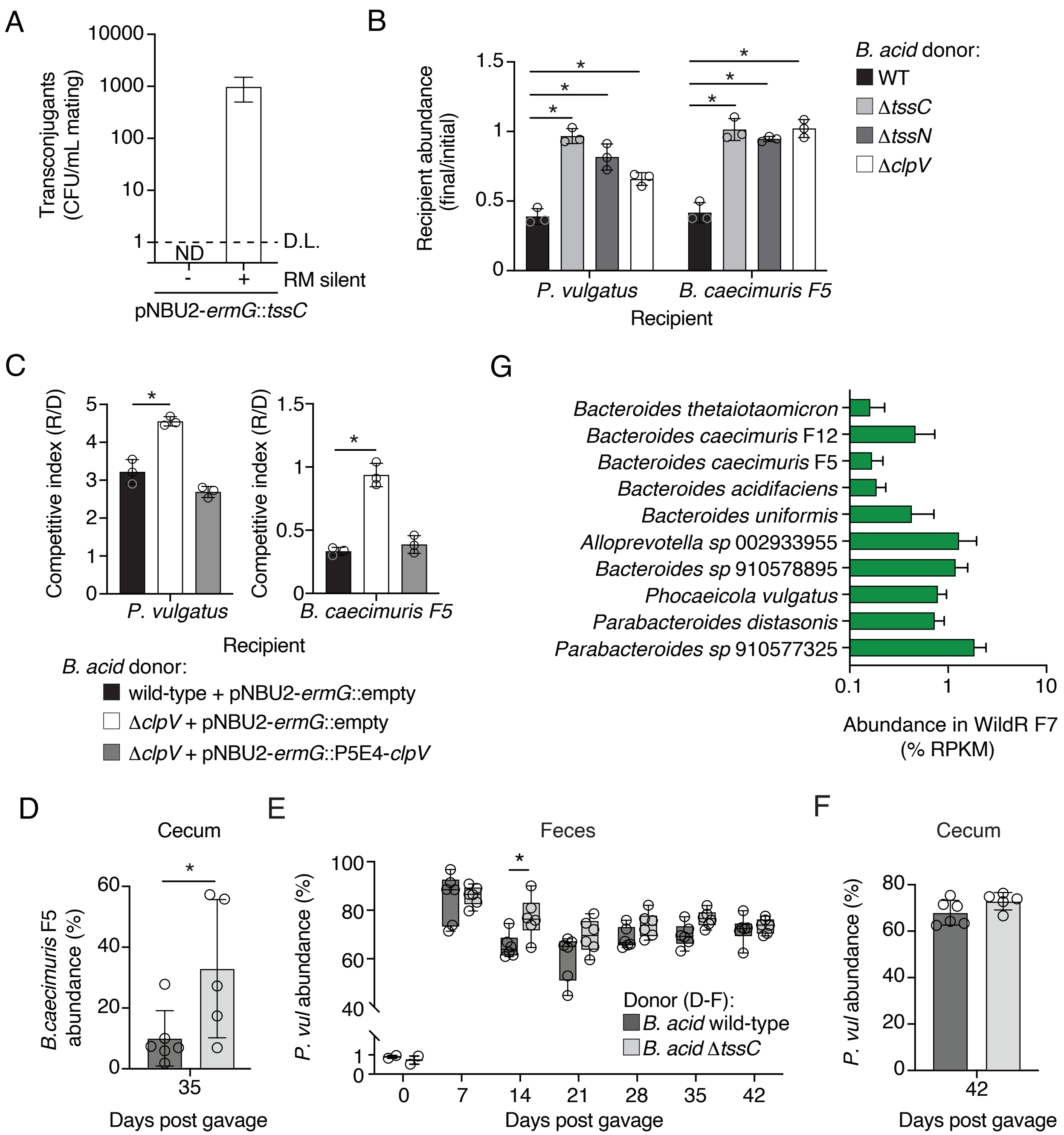

bioRxiv preprint doi: https://doi.org/10.1101/2025.11.29.690828; this version posted December 8, 2025. The copyright holder for this preprint (which was not certified by peer review) is the author/funder, who has granted bioRxiv a license to display the preprint in perpetuity. It is made available under aCC-BY 4.0 International license.

Supplemental Figure 3

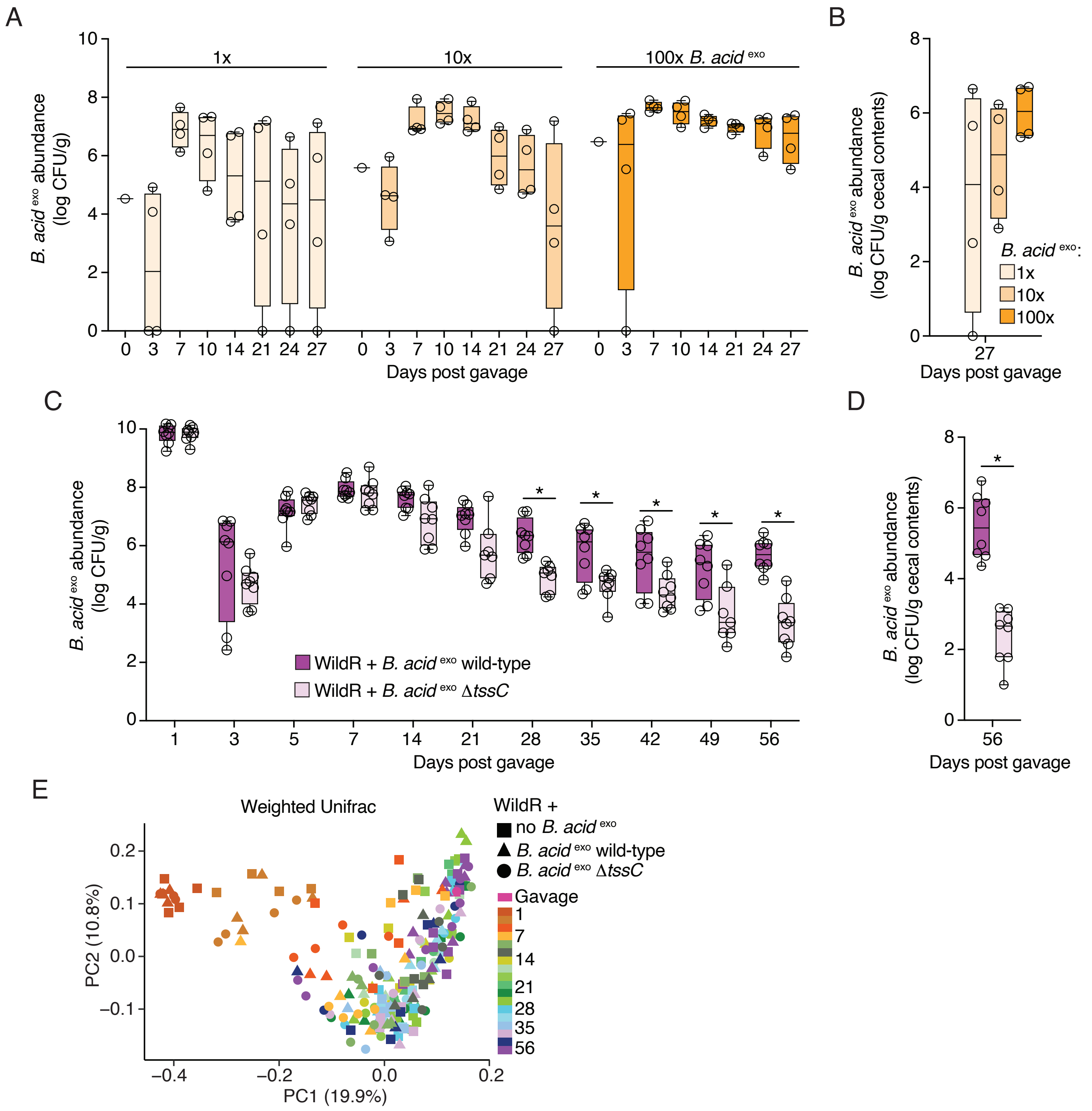

bioRxiv preprint doi: https://doi.org/10.1101/2025.11.29.690828; this version posted December 8, 2025. The copyright holder for this preprint (which was not certified by peer review) is the author/funder, who has granted bioRxiv a license to display the preprint in perpetuity. It is made available under aCC-BY 4.0 International license.

Supplemental Figure 4

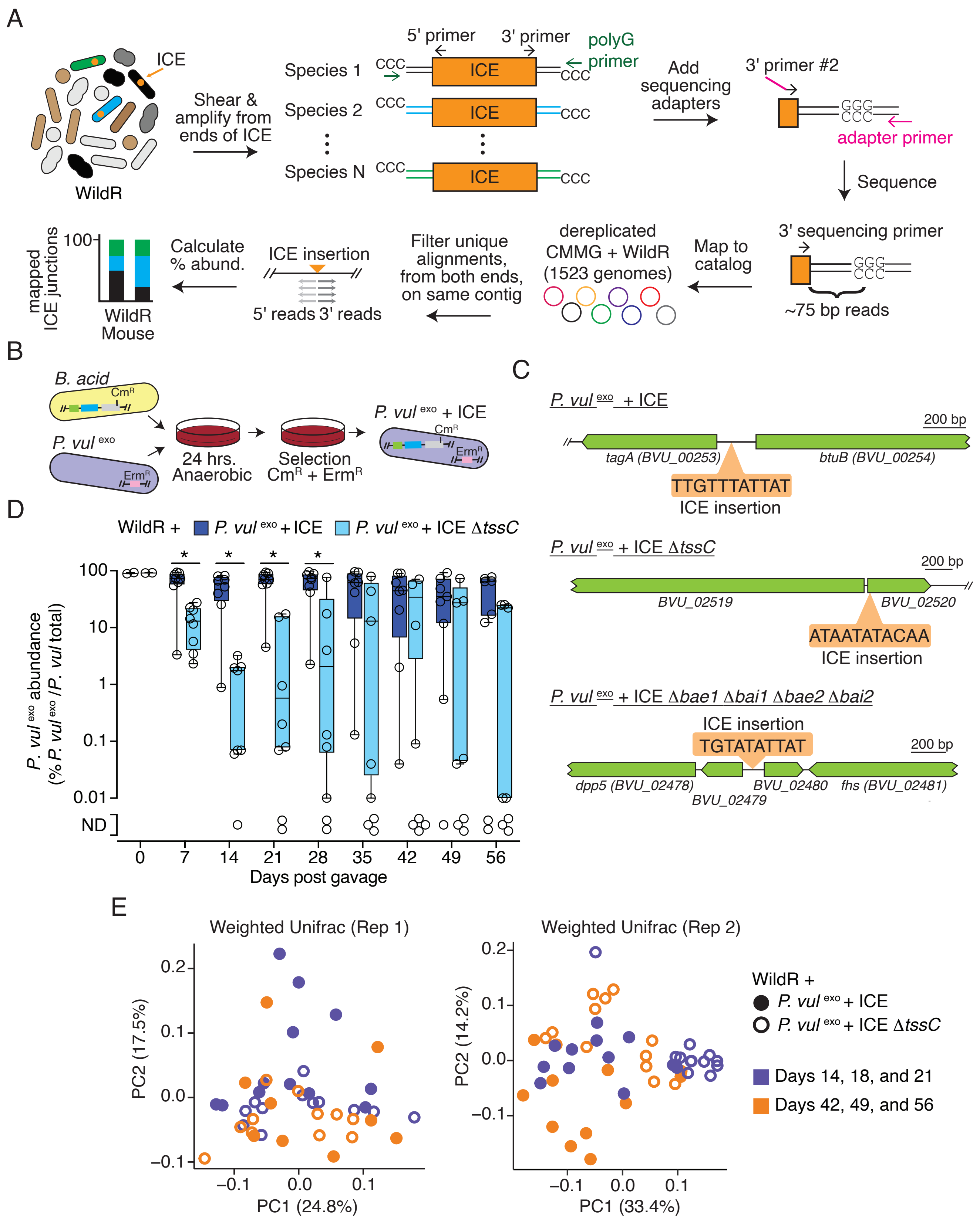

Supplement: Supplement 7 [file NIHPP2025.11.29.690828v3-supplement-7.pdf]
