# Supplementary material for: The type VI secretion system governs strain maintenance in a wild mammalian gut microbiome
Source: bioRxiv. 2025 Dec 3:2025.11.29.690828. Preprint. [Version 2] doi: 10.1101/2025.11.29.690828 (PMC12694586; doi:10.1101/2025.11.29.690828)

bioRxiv preprint doi: https://doi.org/10.1101/2025.11.29.690828; this version posted December 3, 2025. The copyright holder for this preprint (which was not certified by peer review) is the author/funder, who has granted bioRxiv a license to display the preprint in perpetuity. It is made available under aCC-BY 4.0 International license.

Supplemental Figure 1

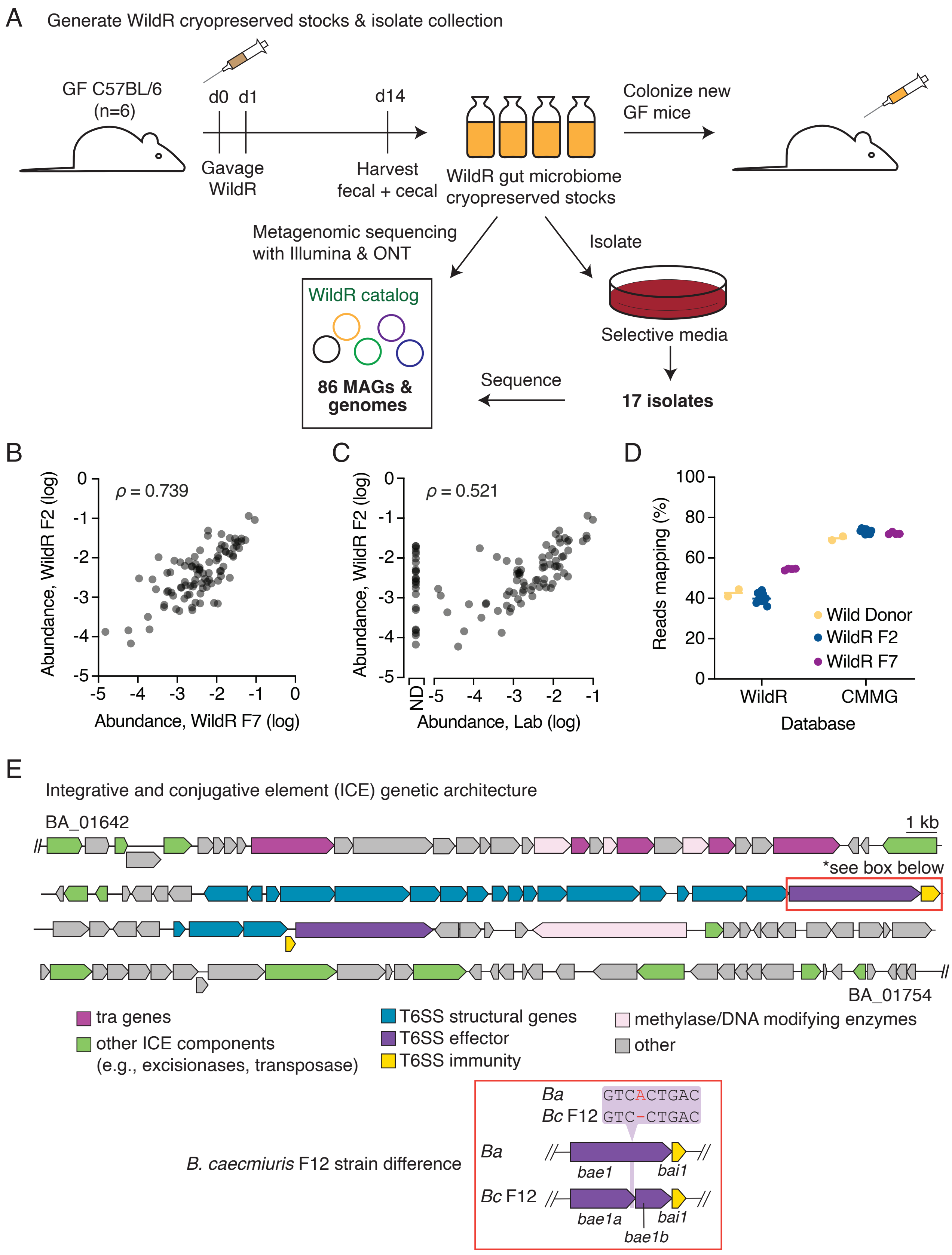

bioRxiv preprint doi: https://doi.org/10.1101/2025.11.29.690828; this version posted December 3, 2025. The copyright holder for this preprint (which was not certified by peer review) is the author/funder, who has granted bioRxiv a license to display the preprint in perpetuity. It is made available under aCC-BY 4.0 International license.

Supplemental Figure 2

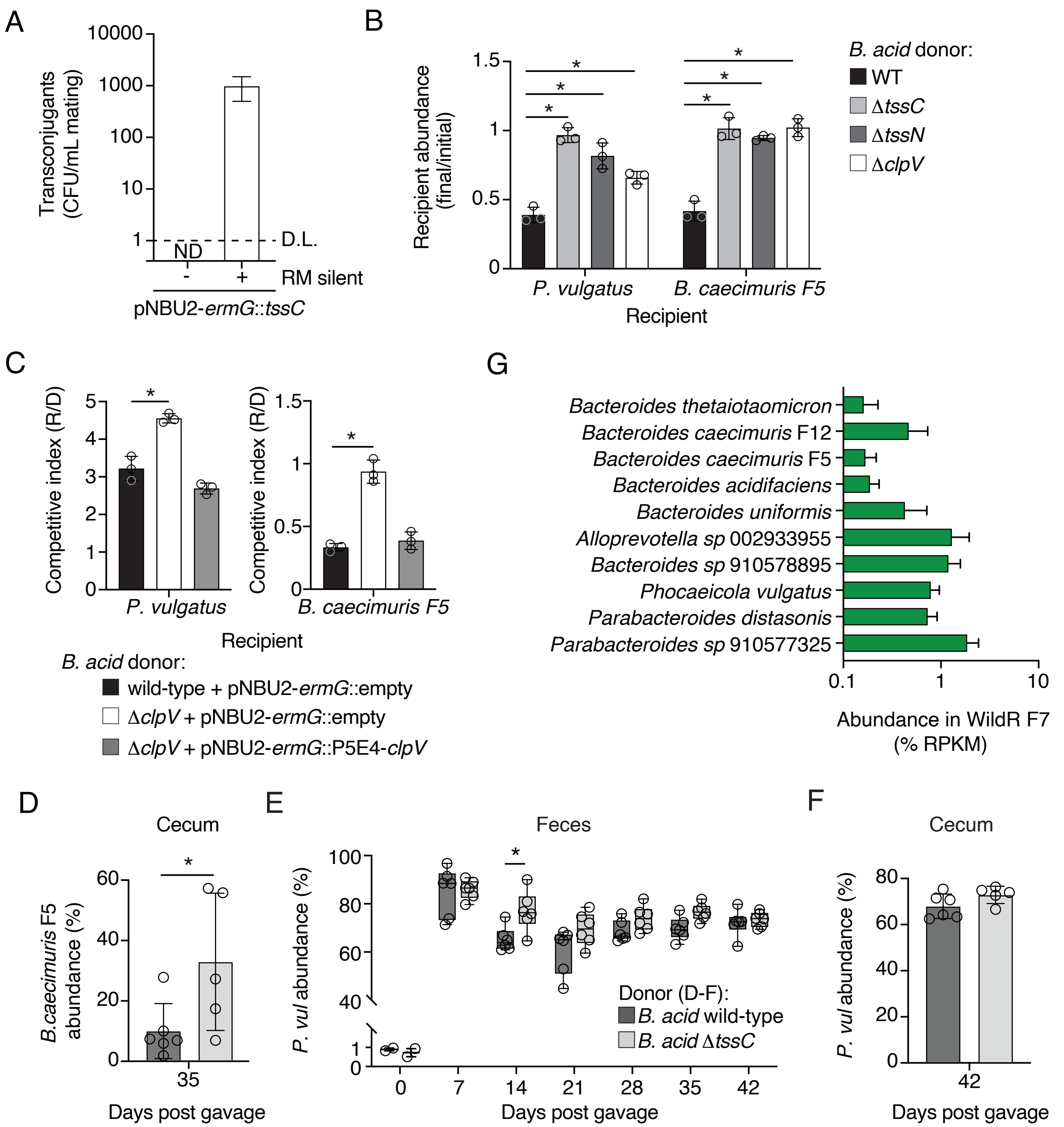

bioRxiv preprint doi: https://doi.org/10.1101/2025.11.29.690828; this version posted December 3, 2025. The copyright holder for this preprint (which was not certified by peer review) is the author/funder, who has granted bioRxiv a license to display the preprint in perpetuity. It is made available under aCC-BY 4.0 International license.

Supplemental Figure 3

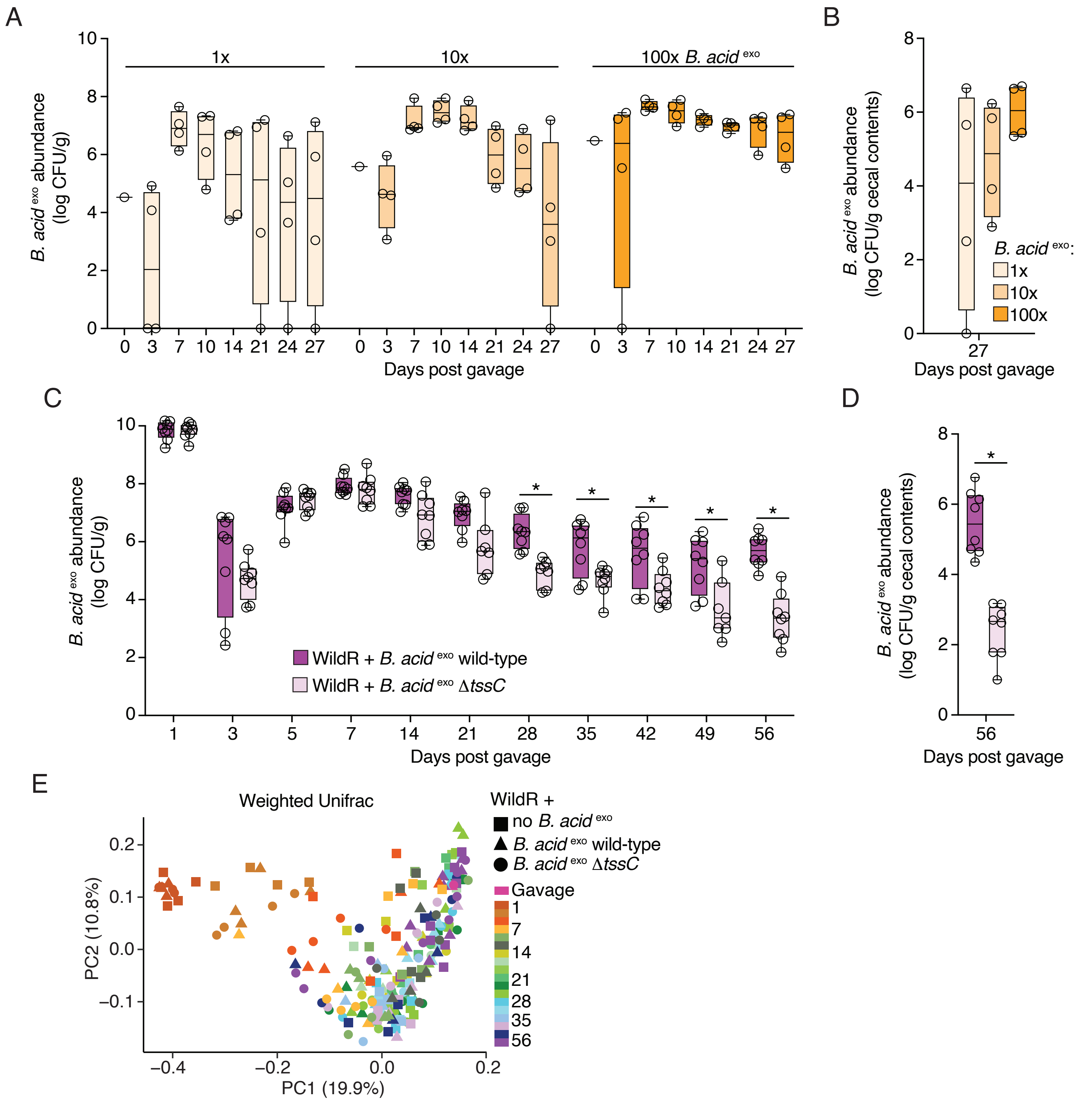

bioRxiv preprint doi: https://doi.org/10.1101/2025.11.29.690828; this version posted December 3, 2025. The copyright holder for this preprint (which was not certified by peer review) is the author/funder, who has granted bioRxiv a license to display the preprint in perpetuity. It is made available under aCC-BY 4.0 International license.

Supplemental Figure 4

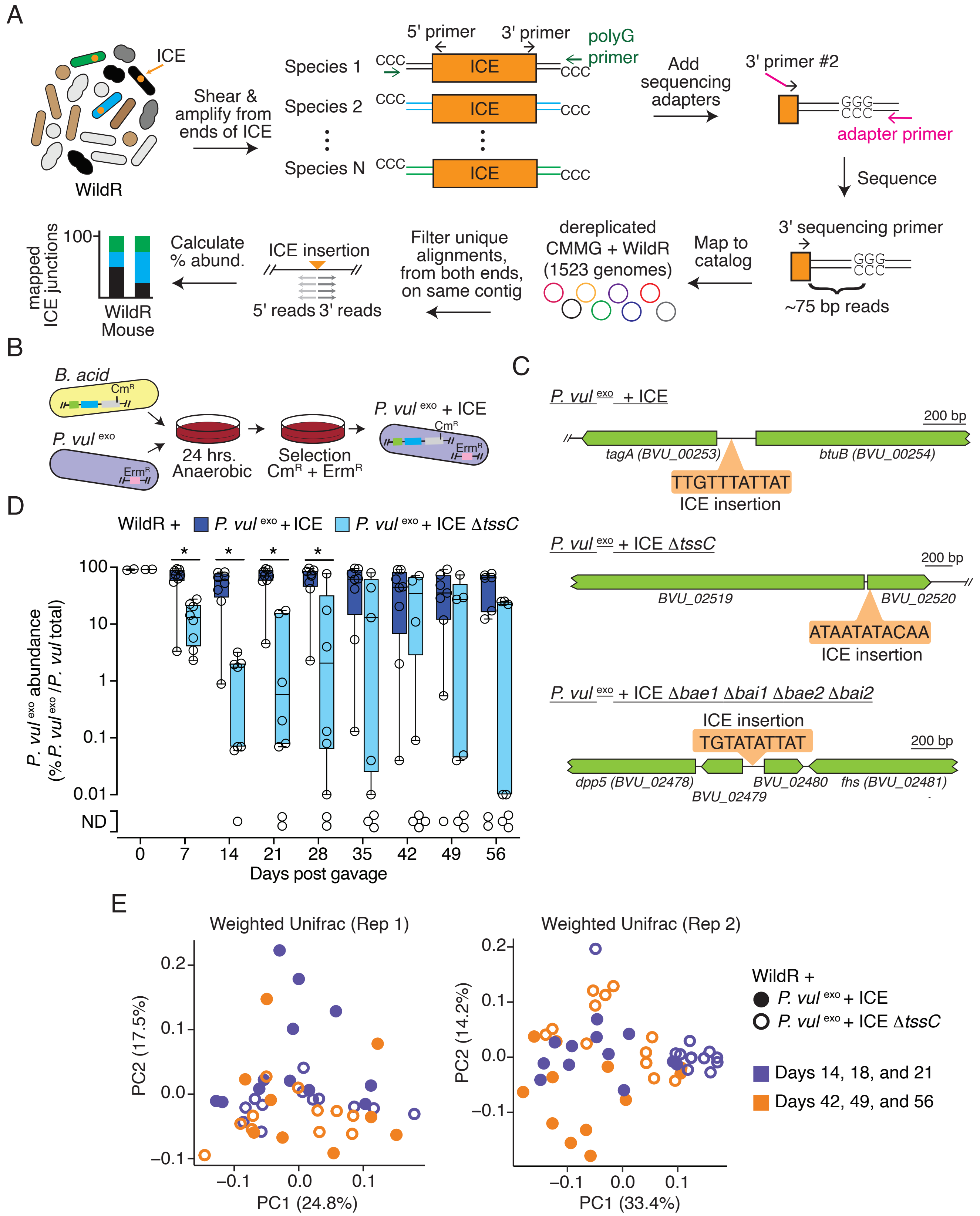

Supplement: Supplementary file 1 [file NIHPP2025.11.29.690828V2-supplement-1.pdf]
